# Sustained Virological Response as a Surrogate Marker for Mortality, Decompensated Cirrhosis, or Hepatocellular Carcinoma in People With Chronic Hepatitis C Virus Infection Treated With Direct-Acting Antivirals: Protocol for a Bayesian and Causal Mediation Analysis

**DOI:** 10.2196/68113

**Published:** 2025-07-09

**Authors:** Kurinchi Selvan Gurusamy, Christian Gluud

**Affiliations:** 1 Division of Surgery and Interventional Science University College London London United Kingdom; 2 Cochrane Hepato-Biliary Group Copenhagen Denmark; 3 The Copenhagen Trial Unit Copenhagen Denmark; 4 Department of Regional Health Research Odense Denmark

**Keywords:** surrogate outcome, chronic hepatitis C infection, HCV, sustained virological response, SVR, direct-acting antiviral drugs

## Abstract

**Background:**

Sustained virological response (SVR) is commonly used as a marker of treatment success in people with chronic hepatitis C virus (HCV) infection. However, there is uncertainty on whether SVR is a validated surrogate marker of successful chronic HCV infection treatment.

**Objective:**

This research project aims to evaluate whether SVR is a good surrogate for all-cause mortality, decompensated cirrhosis, any specific aspect of liver decompensation (jaundice, ascites, hepatic encephalopathy, hepatorenal syndrome, or variceal hemorrhage), or hepatocellular carcinoma in people with chronic HCV infection eligible to receive direct-acting antiviral drugs.

**Methods:**

We will use two ongoing systematic reviews on the effectiveness of direct-acting antiviral drugs in chronic HCV infection as our data sources. The analysis plan is to estimate the regression coefficients or between-studies correlation between SVR and an event using three different Bayesian approaches with OpenBUGS, as outlined in the guidance by the evidence synthesis unit, and estimate the average proportion of the effect mediated through SVR by causal mediation analysis using R.

**Results:**

As of June 19, 2025, the two systematic reviews (one on randomized clinical trials and one on observational studies) on the effectiveness of direct-acting antiviral drugs in chronic HCV infection are ongoing.

**Conclusions:**

We will use the German Institute of Quality and Efficiency in Health Care criterion for surrogacy for cancer, with at least 50% of the treatment effect mediated through SVR, but the information will be reported in a way that allows people to interpret the information using their own criteria.

## Introduction

Hepatitis C is a viral infection of the liver that can lead to liver inflammation [1]. Most hepatitis C virus (HCV) infections extend beyond the first 6 months (acute phase) and become chronic infections [1]. An estimated 50 million people have chronic HCV infections globally [2]. Every year, 1 million people acquire new chronic HCV infections [2].

Major clinical complications of chronic HCV infection include cirrhosis, hepatocellular carcinoma (primary liver cancer), and death resulting from the complications [3]. Decompensated cirrhosis refers to jaundice, ascites, hepatic encephalopathy, hepatorenal syndrome, or variceal hemorrhage resulting from cirrhosis [4]. These complications often result in death [4]. Approximately 240,000 people die from chronic HCV infection globally, mainly due to cirrhosis and hepatocellular carcinoma [2].

Direct-acting antiviral drugs are currently the recommended treatment for people with chronic HCV infection [3]. In our Cochrane systematic review of direct-acting antiviral treatment in chronic HCV infection, sustained virological response (SVR; undetectable HCV RNA after treatment completion) was the commonly used indicator of treatment success in 32 of the 138 randomized clinical trials included [5]. While there is no consensus on the time period after treatment completion during which HCV should be undetectable, the European Association for the Study of the Liver mentions that the relapse of HCV infection beyond 6 months is low, implying that SVR can be considered a stable result after 6 months [3]. Most trials in the Cochrane systematic review used undetectable RNA at 24 weeks after treatment completion as the definition for SVR [5].

The risk of decompensated cirrhosis, hepatocellular carcinoma, and death is lower in people who achieve SVR than in those who do not [6,7]. Therefore, some consider SVR as the fundamental goal in HCV treatment [2,3,8]. Cost-effectiveness analyses (used for determining resource allocation policy) of direct-acting antivirals use SVR rather than direct clinical impact in their decision tree models [9-11], indicating that SVR is the key determinant in cost-utility and plays an important role in resource allocation. On the other hand, only 11 of the 138 trials reported all-cause mortality, while HCV-related morbidity such as decompensated cirrhosis was not reported in any of the trials in the Cochrane systematic review [5]. Therefore, others have questioned whether SVR is a validated surrogate marker of successful treatment of chronic HCV infection; SVR may be a good prognostic marker, but it does not meet the criteria for a surrogate marker, as an increase in SVR does not always translate to an increase in life expectancy, and the correlation between SVR and clinical outcomes does not mean that SVR is a sufficiently good surrogate marker [5,12-15].

This project aims to evaluate whether SVR is a good surrogate for all-cause mortality, decompensated cirrhosis, or any specific aspect of liver decompensation or hepatocellular carcinoma in people with chronic HCV infection eligible to receive direct-acting antiviral drugs.

## Methods

### Data Source

**Figure 1:**
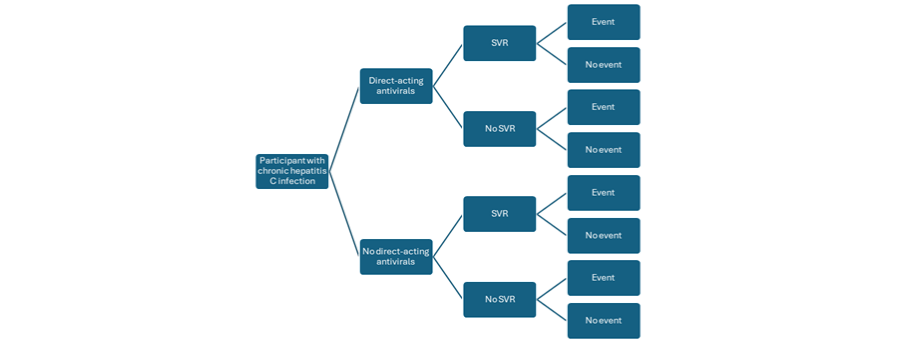
Decision tree. An event is one of the following: all-cause mortality, decompensated cirrhosis, any specific aspect of liver decompensation (jaundice, ascites, hepatic encephalopathy, hepatorenal syndrome, or variceal hemorrhage), or hepatocellular carcinoma. SVR: sustained virological response.

The Cochrane systematic review on the effectiveness of direct-acting antiviral drugs in chronic HCV infection [5] is currently being updated (Professor Goran Bjelakovic, Dr Med Sci, personal communication on June 19, 2025). In addition, a new review on the effectiveness of direct-acting antivirals from observational studies is in progress [16]. As part of these reviews, the information shown in Figure 1 on adjusted treatment effects of the intervention versus the comparator on SVR and event will be collected. In the context of this figure and the protocol, an event is one of the following:

- All-cause mortality
- Decompensated cirrhosis (ie, jaundice, ascites, hepatic encephalopathy, hepatorenal syndrome, or variceal hemorrhage)
- Any specific aspect of liver decompensation (jaundice, ascites, hepatic encephalopathy, hepatorenal syndrome, or variceal hemorrhage)
- Hepatocellular carcinoma

### Analysis

For each outcome, we will evaluate whether SVR is a good surrogate for the clinical outcome. We will reconstruct the individual participant data from the summary data since this is a binary outcome. We will then perform the following to achieve the research objectives.

First, we will estimate the regression coefficients or between-studies correlation between SVR and the event (clinical outcome) using the guidance by the evidence synthesis unit [17] by three different Bayesian approaches using OpenBUGS [18], namely, the standard surrogacy model by Daniels and Hughes [19], bivariate random-effects meta-analysis model [17], and bivariate random-effects meta-analysis in product normal formulation model [20].

We will use three chains and use noninformative priors. We will start with a burn-in of 10,000 iterations and run a further 30,000 iterations to calculate the parameters along with their credible intervals (CrIs). We will check for convergence visually and increase the iterations, using “overrelax” or “thin,” as required to achieve convergence. In addition, we will use the Gelman-Rubin statistic (corrected ratio of the pooled variance across chains and within-chain variance or the “potential scale reduction factor”) to assess convergence [21]. We will consider an upper bound 5% potential scale reduction factor of more than 1.1 for any variable (to be estimated) as indicative of a lack of convergence [22].

For the treatment effects of SVR and the event, which are required to use for these approaches, we will obtain the adjusted odds ratio when available. If not available, we will calculate the odds ratios and 95% CIs of SVR and the event using the total number of participants and the number of participants with SVR or events, respectively, using R (version 4.5.0; R Foundation for Statistical Computing; package: *meta*) [23]. If there are zero events, we will use a correction factor of 0.01 to obtain these measures.

For estimating the correlation between SVR and the event, we will use the Daniels and Hughes [19] model to base our conclusions. We have provided the reasons for doing so in the Discussion section. For the between-studies correlations estimated from the bivariate random-effects meta-analysis model [17] and bivariate random-effects meta-analysis in product normal formulation model [20], we will use the German Institute of Quality and Efficiency in Health Care (IQWiG) criterion for surrogacy, which requires a correlation with the lower limit of the 95% CI to be above 0.85 [24]. We will use 95% CrIs as we will be using Bayesian methods to indicate that SVR is a good surrogate marker for the clinical event. If this cannot be achieved, we will calculate the 80% CrI and see if the lower limit is above 0.85 [24]. If the lower limit of the 80% CrI is above 0.85 but not the 95% CrI, we will consider that SVR may be a good surrogate marker for the clinical event but that there is uncertainty around this conclusion, and further studies are necessary. For the standard surrogacy model by Daniels and Hughes [19], we will consider whether the 95% CrI of the intercept overlaps 0 and the 95% CrI of the association between the treatment differences on SVR and the clinical outcome is positive and does not overlap 0 as criteria for SVR being a good surrogate outcome.

An alternate approach to regression or correlation coefficients when deciding whether SVR is a good surrogate marker is using the models to predict the clinical outcome from the surrogate marker [17]. This approach uses a “take-one-out” approach to predict the treatment effect of the intervention on the clinical outcome in one study based on the observed treatment effect of the intervention on the surrogate marker and meta-analytical estimate of the correlation between the surrogate marker and clinical outcome in the remaining studies [17]. However, we will not use this approach for the reasons described in the Discussion section. We acknowledge that this might introduce uncertainty in validating the model.

Second, we will perform a causal mediation analysis and estimate the average causal mediation effects and the average direct effects using R (package: *mediation*) [25]. We will estimate the average proportion of the effect mediated through SVR and will consider SVR as a surrogate marker if the lower 95% CI of the proportion of the effect mediated through SVR is more than 0.5.

**Figure 2:**
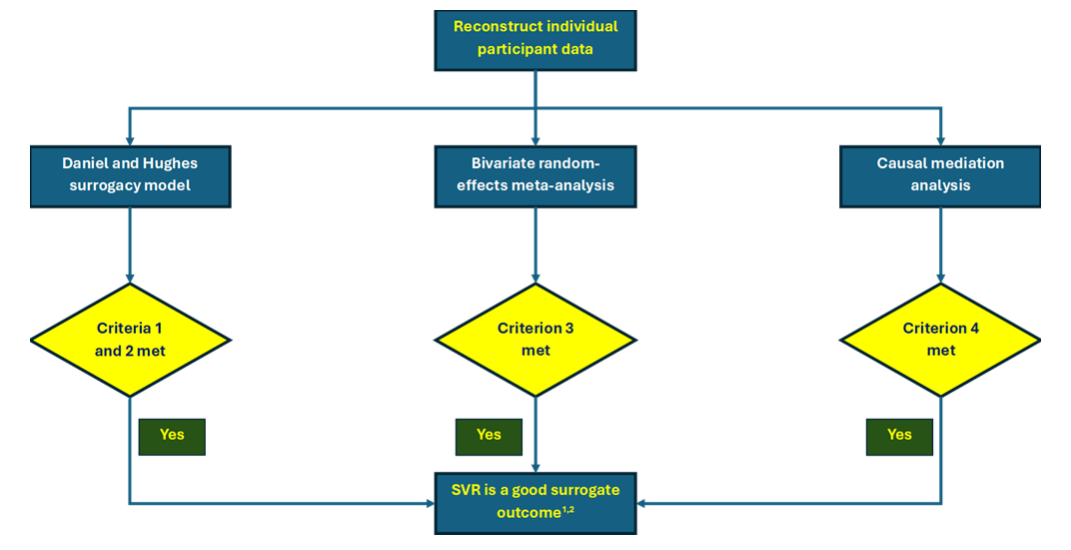
Interpretation algorithm. Criterion 1: 95% credible intervals (CrI) of the intercept overlaps 0. Criterion 2: positive association between the treatment differences in the surrogate and clinical outcomes (95% CrI does not overlap 0). Criterion 3: lower 95% CrI of the between-studies correlations does not overlap 0.85. Criterion 4: lower 95% CI of the effect mediated through the surrogate outcome does not overlap 0.5. If there is a discrepancy between models, we will use the Daniel and Hughes surrogacy model to conclude whether SVR is a good outcome. The between-studies correlations between the treatment differences in the surrogate and clinical outcomes and the proportion of the effect of the direct-acting antivirals will be estimated from the bivariate random-effects meta-analysis model and the causal mediation analysis. For the bivariate random-effects meta-analysis model, if the lower 80% CrI of the between-studies correlations does not overlap 0.85, we will conclude that SVR may be a good surrogate outcome. SVR: sustained virological response.

A summary of our interpretation is available in Figure 2.

### Evidence From Randomized Clinical Trials Compared to That From Observational Studies

We will analyze randomized clinical trials separately from observational studies. If the conclusions based on observational studies are different from those based on randomized clinical trials, we will highlight this difference and indicate the need for corroborating the information from the randomized clinical trials.

### Sensitivity Analysis

If possible, we will perform a sensitivity analysis excluding studies with fewer than 10 years of follow-up because of the duration to develop the clinical complications of chronic HCV infection.

### Ethical Considerations

Since we will be using summary data available from two systematic reviews, an ethics application was not deemed necessary. All randomized clinical trials and some of the observational studies included in the systematic reviews obtained ethical approval before they launched.

### Data Sharing and Reporting

The summary data will be available from the systematic review update, and all authors of the two reviews will be invited to coauthor the results of this research. The data and codes used for this research will be shared via Zenodo. Attempts will be made to publish the report in a journal. If this is not possible, we will share the report of our results through Zenodo.

## Results

As of June 19, 2025, the two systematic reviews (one on randomized clinical trials and one on observational studies) on the effectiveness of direct-acting antiviral drugs in chronic HCV infection are ongoing.

## Discussion

### Justification for Approaches

There is considerable controversy about whether SVR is a good surrogate outcome for clinical outcomes in chronic HCV infection [5,12-15]. However, there has been no previous meta-study evaluating whether SVR is a good surrogate outcome for clinical outcomes in people eligible to receive direct-acting antivirals in the treatment of chronic HCV infection. We will use two different approaches to perform this evaluation. These approaches have been specifically developed to evaluate whether an outcome is a surrogate outcome across a range of medical conditions or whether the effect of an intervention on an outcome is mediated by a surrogate outcome, which is the rationale behind the use of surrogate outcomes. Simpler alternative approaches, such as the association between SVR and the clinical outcomes by odds ratios, establish an association but not a causal association, as a correlation between two variables does not indicate that one variable is a surrogate of another variable [26]. Therefore, we will use the following approaches specifically developed to establish the causal relationship between SVR and clinical outcomes.

In the first approach, for the bivariate random-effects meta-analysis model [17] and bivariate random-effects meta-analysis in product normal formulation model, we will use correlation coefficients to evaluate whether SVR is a good surrogate outcome. There is no consensus on the thresholds used for interpreting correlation coefficients [17]. We will use the IQWiG guidance on surrogate outcomes in oncology [24] to interpret whether SVR is a good surrogate outcome. If the lower limit of the 95% CrI overlaps 0.85, we will check whether the lower limit of the 80% CrI overlaps 0.85. Therefore, we will highlight this additional uncertainty.

Although the limit of 0.85 for the correlation coefficient has been suggested for cancers, we will use this limit for chronic HCV infection also. When there is a high probability of a clinical outcome, one might want to take a more lenient approach to accept surrogate outcomes and treatments that reduce the surrogate outcome when there is a requirement to act early. In the case of cancer, the proportion of people dying within 10 years is 50% across all cancers [27]. Therefore, one might want to take a more lenient approach in approving treatments based on surrogate outcomes. With HCV infection, the proportion of people dying in about 10 years is around 13% [28]. Therefore, one might argue that much stricter criteria should be used for approving treatments for chronic HCV infection based on surrogate outcomes if the treatments are publicly funded.

Others might argue that because of the longer latency period in chronic HCV infections, there are a lot of factors that might change over time, justifying a more lenient approach to approving treatments based on surrogate outcomes. Public funding of treatments is usually based on the cost-effectiveness of a treatment (ie, incremental quality-adjusted life years per incremental costs) [29]. Besides mortality, we will also obtain features of decompensated cirrhosis and hepatocellular carcinoma, which negatively impact longevity and health-related quality of life. Therefore, we will obtain information on both the mortality and the major factors affecting health-related quality of life, the two components of the quality-adjusted life years. A longer period of latency in chronic HCV infections means that the impact of chronic HCV infection on quality-adjusted life years is likely to be less than that of cancer. This goes against using a more lenient approach (for validating and using a surrogate outcome for funding chronic HCV infection treatments) in an already stretched health care system.

To avoid using some new arbitrary threshold criteria, we decided to use similar criteria used for cancer. Furthermore, we will present the correlation coefficients with the 95% CrI (and possibly 80% CrI) along with the data and the codes used for our analyses. If an individual or an organization is interested in using a different threshold for interpreting whether SVR is a good surrogate outcome for clinical outcomes in people eligible for direct-acting antiviral treatment, they can use the data, codes, and results to guide their decision using an alternate threshold.

We anticipate the bivariate random-effects meta-analysis model (standard form) [17] and the bivariate random-effects meta-analysis (product normal formulation) model [20] to result in similar conclusions since we will use the same noninformative prior distributions of parameters in both models and will include only participants without missing data in both analyses. When the prior distributions of parameters are similar and data are available for both outcomes, the bivariate random-effects meta-analysis in product normal formulation model is equivalent to the bivariate random-effects standard form [17]. However, both these models assume that the treatment effect (of the intervention) on the surrogate outcome is exchangeable, while there is no such assumption in the Daniels and Hughes model [17]. When there is a discrepancy between the Daniels and Hughes [19] model and the bivariate random-effects meta-analysis models, we will use the Daniels and Hughes [19] model, as we plan to obtain data from multiple direct-acting antivirals. The treatment effects on SVR with these direct-acting antivirals may or may not be exchangeable.

For interpretation of the Daniels and Hughes [19] model, we will consider whether the 95% CrI of the intercept overlaps zero and the 95% CrI of the association between the treatment differences on SVR and the clinical outcome does not overlap zero as the criteria for SVR being a good surrogate outcome. This is based on the guidance provided by Daniels and Hughes [19] in interpreting the results. The rationale for this approach is as follows. If the entire effect of the treatment on the clinical outcome is mediated through the surrogate outcome, a treatment that does not have any effect on the surrogate outcome must have no effect on the clinical outcome, resulting in the intercept being zero [19]. Furthermore, if the CrI of the association between the treatment differences on SVR and the clinical outcome overlaps zero, this indicates the possibility that there is no association between the treatment differences on SVR and the clinical outcome.

We will not be able to validate the models using the “take-one-out” approach, as this approach is unlikely to be feasible in our studies. This is because the long-term results of direct-acting antivirals are unlikely to be available from randomized clinical trials. In observational studies, the reason for receiving (or not receiving) treatment with direct-acting antivirals may be correlated with the clinical outcome. For example, in one study, the reasons for not receiving direct-acting antivirals included multiple comorbidities, low health literacy, restricted access to hospitals, nursing home residence, and old age [30]; all of these factors are associated with increased risk of death. This means that confounding could be a major reason for an observed association between no treatment and mortality in the observational studies, and reverse causality (ie, the likelihood of the outcome determining whether a patient receives the treatment) may exist in the association between treatment with direct-acting antivirals and increased survival.

Meta-regression is a statistical method that uses study-level characteristics to provide information on whether the treatment effects are affected by the characteristic and is primarily used to explore statistical heterogeneity in meta-analyses [31]. By using confounding factors as the characteristics, it is theoretically possible to calculate the meta-analytical treatment effects across studies, adjusting for the differences in the confounders, for example, the differences in age or proportion of people with major comorbidities between the studies. However, it is unlikely that the adjusted treatment effects of direct-acting antivirals on SVR and clinical outcomes are calculated using the same confounding factors across studies; therefore, it is likely that information on some confounding factors will be missing from some studies. This means it is unlikely that we will be able to use a meta-regression approach to calculate the treatment effects of direct-acting antivirals on clinical outcomes from the treatment effects on SVR, adjusting for the values of the potential confounders. Furthermore, such a meta-regression approach to adjust for study-level characteristics can lead to ecological bias [31,32]. Therefore, we have used the approach of using the regression coefficients and the correlation coefficient to achieve our research objective. However, this is not immune to confounding either, as the regression coefficients may be biased depending on the variables included in the model; this may lead to heterogeneity in the regression coefficients. Furthermore, the regression coefficients do not account for residual confounding. Therefore, the results from the observational studies may be biased.

With our chosen second approach of causal mediation analysis, we will use the lower 95% CI of the proportion not overlapping 0.5 as the criterion for indicating that SVR is a good surrogate marker. This is an arbitrary decision as there is no guidance on the magnitude of the average causal mediation effects that should be considered relevant [33]. We have based the criterion on the rationale that SVR should at least explain most of the effect of direct-acting antivirals on the clinical outcome, if SVR is to be used as the primary objective of treatment or the primary outcome in a clinical trial. Since the 95% CI of the proportion is presented, an individual or organization can use their own criterion for interpreting the information.

As indicated earlier in the Discussion section, there are possible confounding factors explaining the decision to treat (or not to treat) a person with direct-acting antivirals. Therefore, we will analyze the data separately between randomized clinical trials and observational studies.

### Limitations of This Study

We will be relying on data from two systematic reviews (update on the Cochrane review of randomized clinical trials [5] and another systematic review on observational studies [16]). The second author of this study is a coauthor in these studies and will provide information on any deviations from the plans for these systematic reviews due to the peer review. We do not anticipate any major revisions to this research due to the peer review of the two systematic reviews, but we acknowledge the uncertainty of relying on the data.

We have included observational studies in this research because of the limited follow-up time usually found in randomized clinical trials. However, this leads to some limitations in our study. We have discussed one of the limitations of using observational studies, confounding, previously, and there are other limitations.

We will be relying on existing publications. The follow-up in the trials may be different between studies. This can lead to heterogeneity. We will perform a sensitivity analysis, including only studies with at least 10 years of follow-up, to assess the impact of the heterogeneity in follow-ups on the conclusions.

We expect the update of the Cochrane review [5] to identify the randomized clinical trials through the trial registry (because of the mandatory prospective trial registration), thereby allowing an assessment of reporting biases by comparing the registered trials and published trials. However, the other systematic review relies on observational studies; there is currently no mandatory registration of observational studies. Funnel plot asymmetry may help with the identification of publication bias, but the power of the tests used to assess funnel plot asymmetry may be limited with fewer than 10 studies in the meta-analysis [34]. Furthermore, the power is reduced in the presence of heterogeneity, and heterogeneity can be one of the causes of funnel plot asymmetry [34]. All these make it more difficult to identify reporting biases in observational studies.

Because of all these limitations in observational studies, if the conclusions based on observational studies are different from those based on randomized clinical trials, we will highlight the need for corroborating the information from long-term follow-ups in randomized clinical trials using record linkage studies.

We will not be able to validate the models using the “take-one-out” approach. We acknowledge that this might introduce bias, but this bias can be corrected only when randomized clinical trials report long-term clinical outcomes.

## Abbreviations

CrI: credible interval
HCV: hepatitis C virus
IQWiG: Institute of Quality and Efficiency in Health Care
SVR: sustained virological response

## Data Availability

The datasets generated or analyzed during this study will be available in the Zenodo repository.
